# Ischemic stroke destabilizes circadian rhythms

**DOI:** 10.1186/1740-3391-6-9

**Published:** 2008-10-15

**Authors:** He Meng, Tiecheng Liu, Jimo Borjigin, Michael M Wang

**Affiliations:** 1 Department of Neurology, University of Michigan Medical School, Ann Arbor, MI 48109, USA; 2 Department of Molecular and Integrative Physiology, University of Michigan Medical School, Ann Arbor, MI 48109, USA

## Abstract

**Background:**

The central circadian pacemaker is a remarkably robust regulator of daily rhythmic variations of cardiovascular, endocrine, and neural physiology. Environmental lighting conditions are powerful modulators of circadian rhythms, but regulation of circadian rhythms by disease states is less clear. Here, we examine the effect of ischemic stroke on circadian rhythms in rats using high-resolution pineal microdialysis.

**Methods:**

Rats were housed in LD 12:12 h conditions and monitored by pineal microdialysis to determine baseline melatonin timing profiles. After demonstration that the circadian expression of melatonin was at steady state, rats were subjected to experimental stroke using two-hour intralumenal filament occlusion of the middle cerebral artery. The animals were returned to their cages, and melatonin monitoring was resumed. The timing of onset, offset, and duration of melatonin secretion were calculated before and after stroke to determine changes in circadian rhythms of melatonin secretion. At the end of the monitoring period, brains were analyzed to determine infarct volume.

**Results:**

Rats demonstrated immediate shifts in melatonin timing after stroke. We observed a broad range of perturbations in melatonin timing in subsequent days, with rats exhibiting onset/offset patterns which included: advance/advance, advance/delay, delay/advance, and delay/delay. Melatonin rhythms displayed prolonged instability several days after stroke, with a majority of rats showing a day-to-day alternation between advance and delay in melatonin onset and duration. Duration of melatonin secretion changed in response to stroke, and this change was strongly determined by the shift in melatonin onset time. There was no correlation between infarct size and the direction or amplitude of melatonin phase shifting.

**Conclusion:**

This is the first demonstration that stroke induces immediate changes in the timing of pineal melatonin secretion, indicating that cortical and basal ganglia infarction impacts the timing of melatonin rhythms. The heterogeneous direction and amplitude of melatonin shifts suggests that the upstream regulation of hypothalamic timekeeping is likely anatomically diffuse and mechanistically complex. Finally, our study exemplifies the use of pineal microdialysis to evaluate the effect of neurological diseases on circadian function.

## Background

Stroke is a leading cause of death and disability world-wide. While the immediate consequences of stroke include permanent cognitive deficits, paralysis, visual impairment, and sensory disturbances, stroke also results in long term dysregulation of sleep and mood, which may be equally disabling. The influence of ischemic stroke on circadian rhythm regulation, which is strongly linked to sleep and mood, may thus potentially influence long-term recovery in stroke patients.

In humans, the daily expression of a multitude of physiological variables is suppressed by ischemic stroke [1-8], but limited work has been done to establish whether these effects reflect true disruption of the circadian timing system. To address the status of the central circadian pacemaker, other investigators have measured melatonin after stroke [9]. Beloosesky et al. [10] sampled urinary melatonin metabolites every four hours and found that rhythms persisted in all stroke patients, but the phase was delayed by about 4 hours during the first day in patients with extensive brain injury; the delay was not apparent 10 days after stroke. Fiorini et al. [11] measured urinary melatonin every 12 hours after stroke and demonstrated decreases in stroke patients that persisted for two weeks. The precise timing of melatonin secretion could not be determined in this study. Pang et al. [9] demonstrated that intracerebral hemorrhage in a variety of locations inhibits the production of melatonin measured by venipuncture. The lack of longitudinal data in human subjects makes it difficult to determine whether stroke induces changes in circadian timing. In addition, the interpretation of these studies is difficult, since standard medical care of acute stroke patients by necessity requires nocturnal disruption that may suppress melatonin and falsely imply changes in circadian timing.

To overcome limitations of clinical studies, a small number of experimental systems have been used to study the effect of stroke on circadian regulation. Work in mice has shown that sleep [12] is inhibited by ischemic stroke. Spontaneously hypertensive stroke prone rats, which spontaneously undergo both hemorrhagic and ischemic stroke, exhibit blunting of heart rate and blood pressure rhythms, but changes in circadian timing were not clear [13]. The circadian variables in these studies have limited time resolution and thus are insufficient to prospectively quantify circadian phase changes.

Pineal microdialysis offers an exceptional technique for measuring quantitative changes in circadian melatonin secretion [14]. This method enables real time analysis of a single individual, and therefore can prospectively measure quantitative circadian responses to environmental changes, pharmaceuticals, and potentially disease states. In addition, the precise and sensitive measurement of pineal melatonin permits the quantitative determination of melatonin phase with up to 10-minute resolution. Pineal melatonin is the most accurate marker of the central circadian clock [15,16] and is not subject to pathological masking that could be affected by the neurological compromise (eg. suppression of motor activity secondary to hemiparesis, abulia, or ataxia after stroke). In this report, we applied pineal microdialysis to determine whether ischemic stroke in rats alters the circadian rhythm.

## Methods

### Animals

All experiments were performed in accordance with NIH guidelines and approved by the University of Michigan Committee on Use and Care of Animals. Adult male Wistar rats (300 g) (Harlan) were housed at 20 to 25°C under LD 12:12 h conditions, with ad libitum food and water. Illumination levels were 300–400 lux at cage level.

### Microdialysis probe implantation

Surgical placement of microdialysis probes was performed as described [14]. Briefly, following deep anesthesia, the animal's head was shaved and positioned in a stereotaxic instrument with the head flat. Two small burr holes were created on both sides of the skull. A custom linear microdialysis probe was inserted carefully through the burr holes into the brain tissue containing the pineal gland and was connected with dialysis tubing at both ends. Following surgery, animals were housed individually and allowed to recover for 16–24 h before microdialysis.

### Sample collection and analysis

Following recovery, animals were transferred to dialysis chambers where their pineal glands were continuously perfused with artificial cerebral spinal fluid at a low (2 ul/min) rate via inlet tubing connected at one end of the microdialysis probe. The outlet was connected to a sample loop of an HPLC sample injector, which automatically injected the collected pineal dialysates into an HPLC column at 10 min intervals. Melatonin in the dialysates was resolved on a C18 reverse phase column and flow through was analyzed by a fluorescence detector controlled by Shimadzu software.

### Middle cerebral artery occlusion

Animals were subjected to ischemic stroke by filament occlusion of the middle cerebral artery (MCA) after baseline melatonin monitoring. The stroke model has been described before [17,18]. Animals were taken off of melatonin monitoring for the stroke surgery 3–6 days after the initiation of on-line microdialysis during which time baseline melatonin profiles were obtained. We occluded the right MCA for two hours using an intralumenal filament and then removed the filament to allow reperfusion. We placed the animals back online for pineal microdialysis and continued melatonin monitoring for up to seven days or until the animal appeared in extremis (whereupon it was euthanized). At the end of the monitoring period, animals were euthanized and brains were cut into 2 mm thick sections and analyzed by 2% 2,3,5-triphenyltetrazolium chloride (TTC) staining to quantify infarct volumes; TTC stains vital tissue red while dead tissue remains unstained. Infarct volumes were determined by analysis of imaged brains using NIH Image software. Sham surgery involved insertion of the filament into the carotid artery without advancing the suture to the MCA bifurcation.

### Statistics

To determine the level of statistical significance of circadian changes, we first calculated the average absolute deviation from the baseline melatonin onset (MT-on) for each day after stroke for each animal. We used the average of pre-stroke MT-on times as the baseline. We then computed the absolute value of the difference between MT-on and the baseline for each day that the animal was monitored. Finally, we calculated the average value of the absolute shift over the entire duration of the monitoring period. This metric accounts equally for both delays and advances in timing. The same calculation was performed for sham animals after control surgery. The mean of the average daily deviation for all animals in each group was compared between MCA occlusion (MCAo) and sham groups using a two-tailed t-test.

## Results

**Figure 1:**
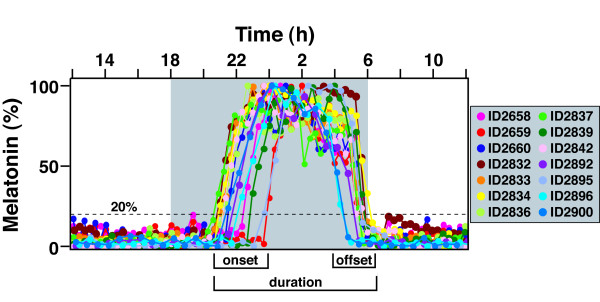
**Baseline melatonin profiles for rats prior to MCAo**. Melatonin profiles were obtained for all animals prior to MCAo to determine baseline timing. Measurements were made for up to six days, and, in all cases, the daily melatonin timing was stable. Therefore, only a single day's tracing of each individual is shown in this composite illustration. Animals exhibit a wide range of timing profiles. There is a broader range of MT-on times compared to MT-off times. The melatonin duration is defined as the time between the MT-on and MT-off. The MT-on is defined as the time when melatonin rises to 20% of the daily maximum (set to 100%). The MT-off is defined as the time when the melatonin level falls to 20% of the daily maximum. The gray shaded area represents time of lights off.

Prior to stroke, we implanted Wistar rats (n = 14) with pineal microdialysis probes and determined baseline melatonin secretion patterns. Since this is an outbred population, we observed a range of MT-on and melatonin offset (MT-off) times (Figure 1), defined by the interval between time of dark onset and time when the melatonin rhythm reached 20% of its nightly maximum. The variation of MT-on and MT-off within the cohort has been described previously [19]. Melatonin duration was calculated as the time difference between MT-on and MT-off. Although there was variation within the cohort of circadian profiles, individual rats prior to stroke showed virtually no day-to-day variation in MT-on, MT-off, or melatonin duration over more than three days of baseline observation, as described previously (14).

**Figure 2:**
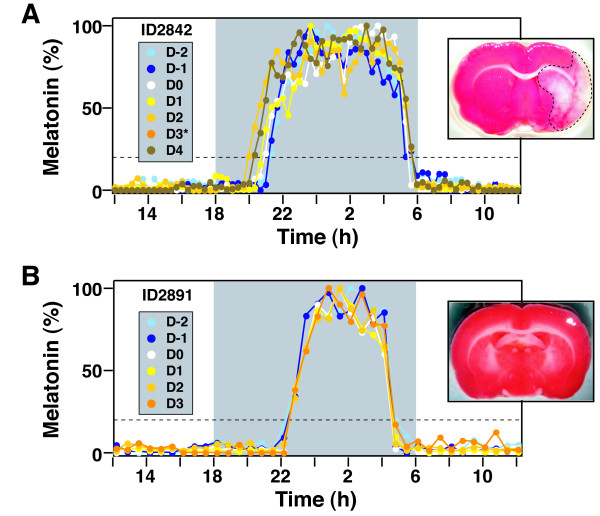
**Effect of MCAo on rhythmic secretion of melatonin**. Melatonin secretion was measured in animals before and after surgery. Representative animals undergoing MCAo (A) and sham surgery (B) are shown. Tracings represent daily melatonin profiles superimposed upon the time of the day, with the gray shaded area representing lights off. Each day is demonstrated in a different color. D-2 and D-1 are two and one day before surgery. D0 is the day of the surgery, which was performed in the daytime. Days one to four (D1 to D4) are post-operative days. MCAo caused an advance of MT-on that persisted through four days of monitoring (A). MCAo slightly delayed the MT-off. Due to technical reasons, the data for the third postoperative day was lost (D3*). TTC stained brain sections are displayed to demonstrate that MCAo induces cortical and subcortical infarction. Sham surgery (B) did not affect MT-on or MT-off timing and did not cause brain infarction.

MCAo surgery was then performed on these animals. All surgeries were initiated between 12:15 h and 14:34 h during the day. Rats were then placed back on microdialysis to measure post-surgical circadian melatonin profiles. We could not record melatonin secretion from one animal, and it was excluded from analysis. Two animals did not show changes in melatonin secretion after MCAo. Eleven of the remaining 13 rats exhibited temporal shifts in melatonin secretion, beginning with the first night after MCAo (Day 0); a representative rat is shown in Figure 2A. These rats demonstrated clear brain infarctions of the cortex and basal ganglia, demonstrated by TTC staining (Figure 2A).

We also performed sham MCAo (control) surgery on six rats that were analyzed by pineal microdialysis. These rats had pre-surgical melatonin profiles that were similar to the rats with MCAo. All six rats exhibited identical timing of melatonin secretion before and after control surgery; a representative animal is shown in Figure 2B; as expected, TTC staining of control brains showed no infarction.

**Figure 3:**
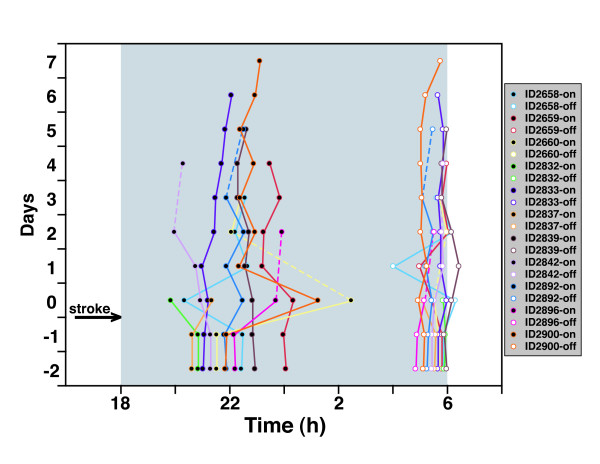
**Heterogeneous responses of melatonin timing after MCAo**. MT-on and MT-off times were obtained for each day of the experiment and are displayed in a composite timeline. Individual rats' melatonin profiles are depicted by unique colors. The left side shows MT-on tracings with black-filled circles, whereas the right side shows MT-off tracings with white-filled circles. As before, the day of MCAo was defined as Day 0. Due to technical difficulty, data was not available for some days for specific animals (dotted lines were used when days were skipped).

**Figure 4:**
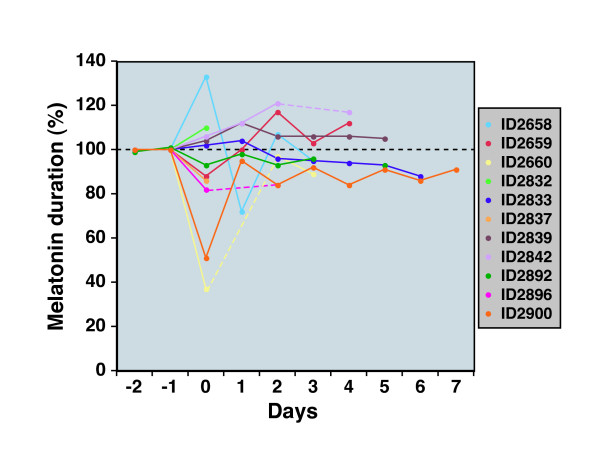
**Effect of MCAo on the duration of nightly melatonin secretion**. Melatonin duration was calculated by computing the time between MT-on and MT-off for each day of the experiment. Since melatonin duration differs among individuals in the cohort, the melatonin duration was therefore normalized to the stable pre-stroke melatonin duration (set to 100%). Melatonin duration plots are displayed for each individual in a different color. As before, the day of MCAo was defined as Day 0. Dotted lines are used to signify days when data were not available for technical reasons.

**Figure 5:**
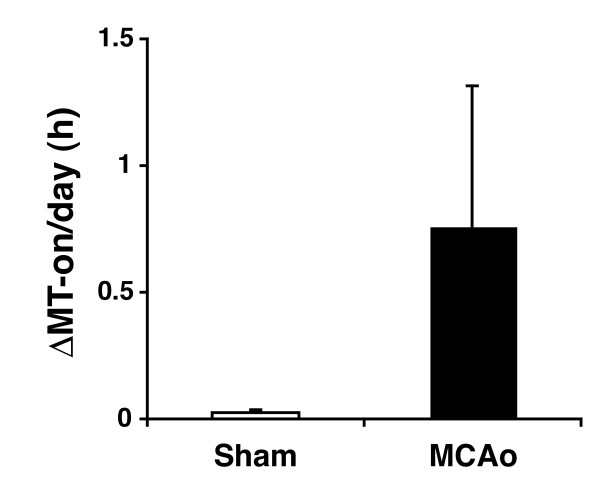
**Effect of MCAo on the shift of nightly melatonin onset**. The impact of stroke on the melatonin rhythm for each animal was calculated by averaging the absolute daily melatonin onset shift (relative to pre-stroke baseline); see methods for a discussion of this calculation. The averages of these values determined for all animals with MCAo or sham surgery are displayed in bar graph format. The difference between average melatonin onset shift in the MCAo group was statistically significant relative to the control group (p < 0.02).

We examined the entire cohort of animals that exhibited melatonin shifts to attempt to identify trends in circadian behavior. Figure 3 shows the MT-on and MT-off profiles for this set of rats. Surprisingly, the pattern of circadian phase shifts in melatonin secretion showed a heterogenous amplitude and direction of change. In general, however, the MT-on shifts were more pronounced than MT-off shifts. The duration of melatonin secretion also exhibited significant differences after stroke in these animals (Figure 4). The changes in melatonin duration varied in direction and amplitude. When daily shifts of MT-on were averaged following stroke surgery (a measure of deviation from baseline – see methods), we observed a significant difference (p < 0.02) between sham and MCAo treated animals (Figure 5).

**Figure 6:**
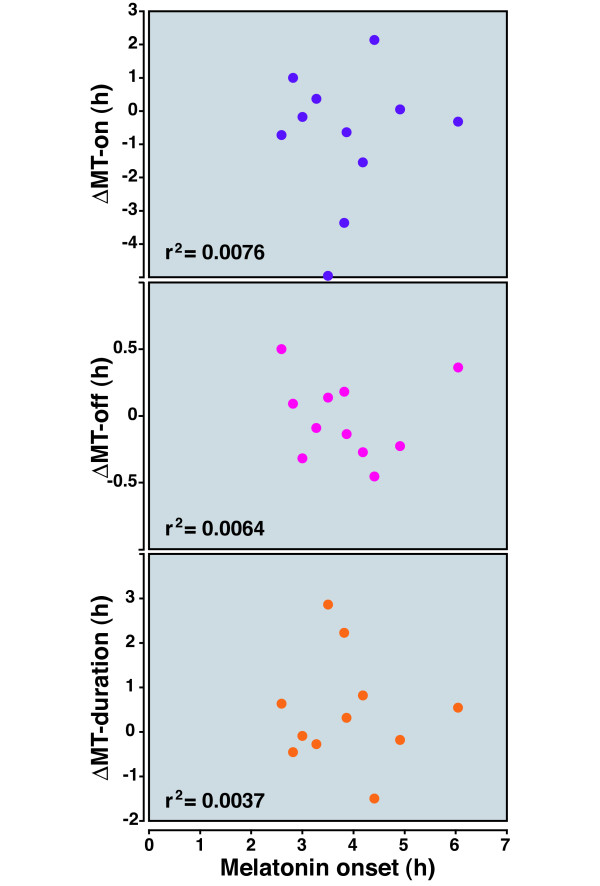
**Lack of relationship between the phase angle of MT-on and the circadian response to MCAo**. As seen in Figure 1, rats demonstrate a wide range of baseline phase angles of MT-on. We explored the relationship between the phase angle (on the day of the stroke; shown on the X-axis) of MT-on, MT-off, and melatonin duration, relative to the baseline values before MCAo. The r^2 ^values are shown; no relationship was found.

As discussed above, the pre-stroke phase angle of MT-on displayed variations within the cohort, indicating that individual animals exhibited unique circadian parameters. However, individual variations in MT-on time did not correlate with the direction or amplitude of circadian shifts after stroke (calculated on the initial day after the stroke; Figure 6).

**Figure 7:**
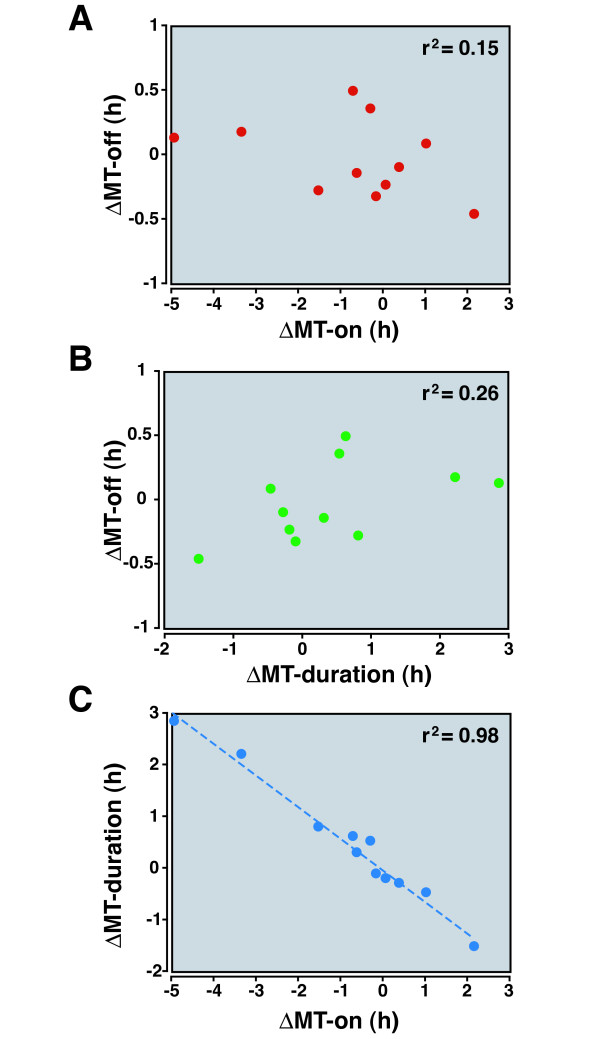
**Change in melatonin duration is related to the change in MT-on after MCAo**. Results from the day of the MCAo were examined here. Data displayed include the change in MT-on, MT-off, and melatonin duration relative to respective pre-MCAo values. (A) There was no relationship between shifts in MT-on and MT-off after stroke. Animals exhibited all four possible combinations of directions of shift. (B) There was no relationship between the shifts in MT-off and melatonin duration. (C) Change in the melatonin duration after stroke was strongly linked to the shift in MT-on. The r^2 ^values are shown.

The direction of MT-on and MT-off shift (on the first night after the stroke) demonstrated no correlation with each other (Figure 7A). Roughly equal numbers of rats experienced an advance in MT-on compared to delays, and roughly half experienced advances in MT-off compared to delays. We observed all four combinations of MT-on/MT-off patterns (advance/advance, advance/delay, delay/advance, and delay/delay). The change in MT-off was also not associated with melatonin duration (Figure 7B).

Interestingly, the initial change in melatonin duration was strongly linked to the initial change in MT-on after stroke (Figure 7C). Thus, the change in melatonin duration after stroke could be predicted by observing MT-on alone. This relationship held true several days after the stroke, as well (see below).

**Figure 8:**
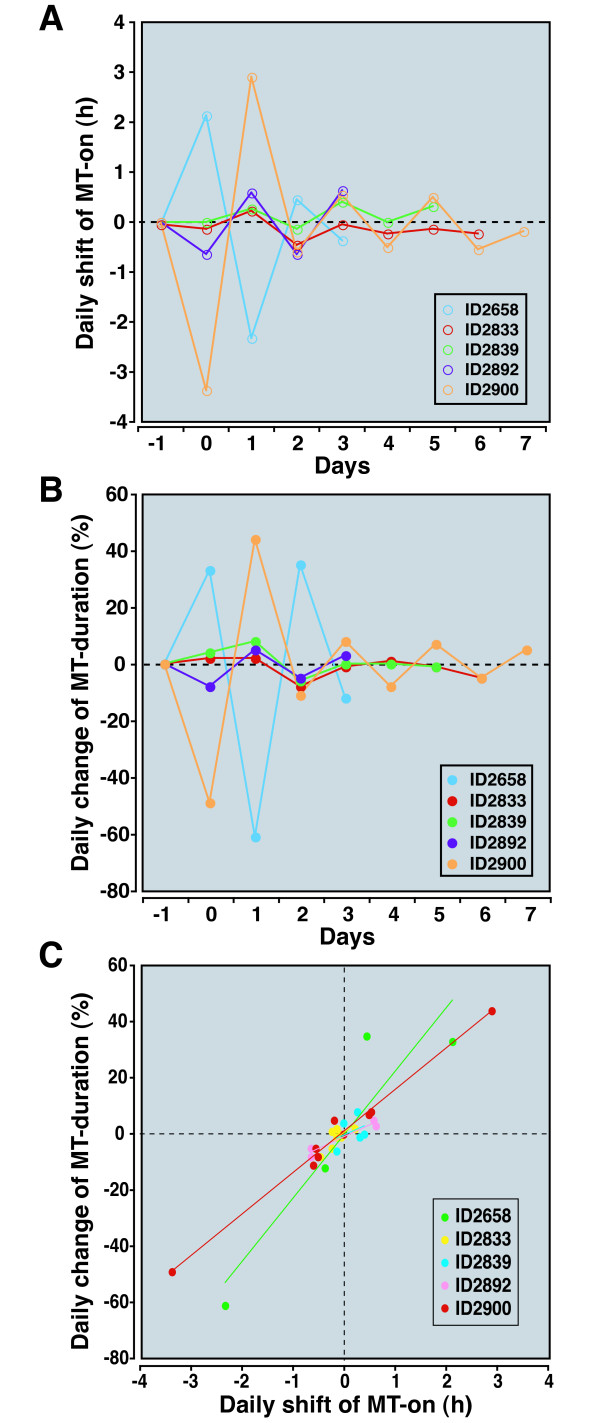
**Day to day changes in MT parameters after stroke**. The day-to-day changes in MT-on and melatonin duration were defined as the change in melatonin timing compared to the time on the previous day. As expected, on Day -1, there is no shift since the melatonin rhythm is stable. However, on Day 0 (the day of the stroke) there is a change in the melatonin timing compared to before stroke. Only five animals, represented by different colors, were used for this analysis. Other animals in the study could not be used because of gaps in data. (A) MT-on changes markedly in most animals from day-to-day. In all animals, there is a remarkable oscillation of the daily changes in melatonin timing. In general, the amplitude of the oscillation decreases over time. (B) A similar trend is seen with the daily change in melatonin duration. (C) There is a strong relationship between the daily shift in MT-on and melatonin duration among most animals. The r^2 ^coefficients (for each animal) were: 0.85 (ID2658), 0.56 (ID2833), 0.15 (ID2839), 0.92 (ID2892), and 0.99 (ID2900).

Due to early death (ID2832 and ID2837) and technical reasons (ID2660, ID2842, and ID2896), we were not able to quantify MT-on and MT-off for every day in every animal. However, in animals for which we had daily data for extended periods, the MT-on and melatonin duration demonstrate highly dynamic day-to-day changes after stroke (Figures 8A and 8B); the change in MT-on and melatonin duration from day-to-day exhibited an alternating pattern, seeming to oscillate after stroke, albeit with progressively decreasing amplitude. The waning of day-to-day changes suggests that over time, the melatonin onset and duration are reaching equilibrium at a new set point. As on the first day after MCAo, MT-on and melatonin duration changes over the course of a few days correlate well with each other, indicating that the main determinant of melatonin duration changes after stroke is the MT-on shift (Figure 8C).

**Figure 9:**
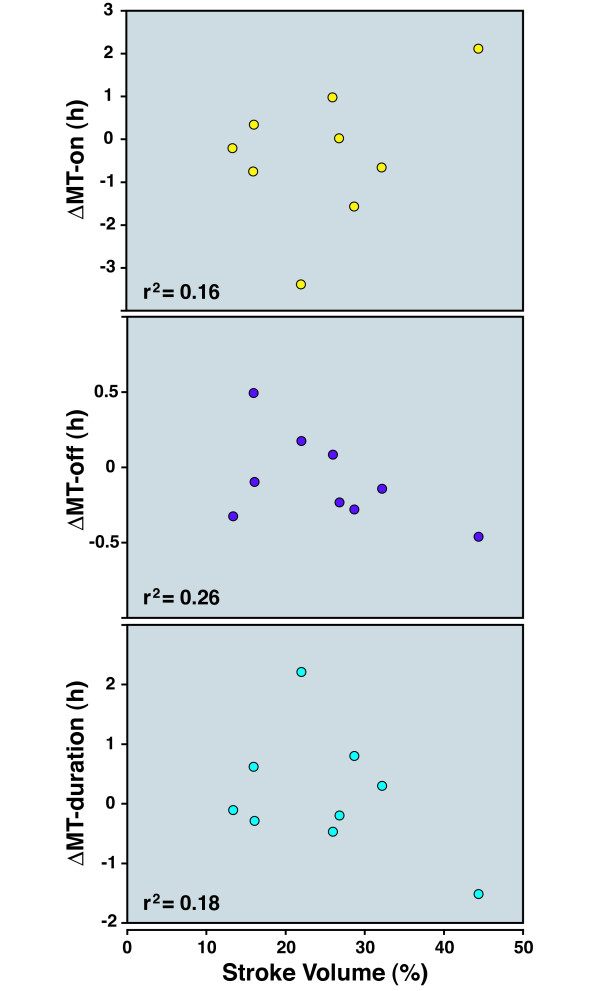
**Infarct volume and its relationship to shift in MT timing**. Infarct volumes for all animals undergoing MCAo were computed. Each rat's infarct volume was plotted against the Day 0 shift in MT-on, melatonin duration, or MT-off relative to the baseline time before stroke. There was no linear relationship between infarct volume and any of the quantitative measures of circadian changes. The r^2 ^values are shown; no relationship was found.

To determine whether circadian melatonin shifts were related to the degree of infarction, we computed the area of brain infarction using established morphometric methods. The range in infarction volume was typical of stroke studies (30% +/-15%). We could not identify a correlation between the degree of damage and either the direction or the amplitude of initial changes in MT-on, MT-off, and melatonin duration (Figure 9).

Melatonin production in the pineal gland is controlled by a number of brain structures that include the SCN, paraventricular nucleus of the hypothalamus (PVN), and intermediolateral nucleus of the spinal cord (IML). In addition, the thalamic intergeniculate leaflet (IGL) [20] modulates SCN activity, which may affect melatonin output. Additionally, structures such as caudate putaman (CPu) and parietal cortex are involved in locomotor rhythm generation that can be dissociated from the SCN signals [21], and may be affected in animals after stroke. To determine whether specific regions affected by MCAo were responsible for melatonin rhythm disruption, we examined lesion sites of MCAo-treated animals. Of the animals that exhibited shifts in melatonin rhythms, only one showed injury near the SCN and only three animals showed infarctions of the IGL. None of the animals had injury to the PVN or IML. This indicates that injury to these structures cannot account for the change in melatonin timing after stroke. On the other hand, all animals had significant injury to the CPu, and all but two animals had cortical injury. Thus, CPu stroke is sufficient by itself to disrupt normal melatonin rhythms.

## Discussion

This is the first study to our knowledge that demonstrates an effect of stroke on diurnal melatonin secretion in experimental animals. Our experiments unequivocally demonstrate that ischemic strokes in rats disrupt the timing of melatonin onset and/or offset, which are the most consistent and direct markers of circadian timing. We also demonstrate that pineal microdialysis is a highly sensitive method for the study of modulation of the circadian pacemaker by disease states.

Our prospective study design using pineal microdialysis overcomes methodological limitations encountered in human studies and in animal studies that utilize other circadian markers. Human studies are confounded by the inability to measure pre-stroke circadian parameters, heterogeneity of stroke location, pre-existing medical conditions, medications, neurological disability and the unavoidable environmental variation required for medical care. As discussed above, melatonin is superior to wheel running activity, core body temperature, and heart rate as a marker for animal circadian timing; in addition, melatonin is commonly used as a clock marker in human studies [15].

In agreement with the human study by Beloosesky et al[10], we show that in the acute phase of stroke, melatonin rhythms are still present, but that the phase of melatonin rhythms is shifted. Importantly, our experiments give us added temporal resolution to quantify circadian changes and to observe unique patterns of clock plasticity after stroke. Our ability to examine individual melatonin profiles reveals that there is dramatic heterogeneity in the response of melatonin after stroke that cannot be detected in human cohort observations.

We detected oscillatory behaviors in the MT-on alteration and the melatonin duration change, which suggests that a neuronal mechanism of non-pineal origin could be responsible for reestablishing the steady state after ischemic stress. The oscillatory melatonin rhythm demonstrates that in the days after stroke, the circadian rhythm is unstable. However, the oscillations dampen over days, suggesting that the rhythm will ultimately reach an equilibrium state with a stable melatonin rhythm similar to the pre-stroke rhythm. Our finding that stroke induces changes in melatonin secretion for several days differs from the previous human studies [10]; we believe that our results may be due to our ability to sample melatonin with higher temporal resolution (20 minute vs. 240 minute increments) directly from the pineal gland (versus a urinary metabolite).

We were surprised by the heterogeneity of melatonin responses to stroke. Our data shows that the heterogeneity is not related to a pre-existing circadian phenotype and is not a result of differential damage to the hypothalamus, which is spared in this model of stroke.

There may be several explanations for the heterogeneous effects of stroke on circadian rhythms. We speculate that diffusely distributed cortical and subcortical neurons exert distinct effects on different sets of hypothalamic neurons. Broadly distributed circadian controlling neurons may be responsible for the highly variable changes in melatonin secretion due to the stochastic nature of stroke injury, which is in the same general territory, but varies subtly from animal to animal. Alternatively, since stroke causes massive releases of glutamate into the brain, it is possible that glutamate diffusion into the hypothalamus results in the disruption of hypothalamic clock neurons. The regional spread and timing of glutamate release into the hypothalamus may differ between individuals, resulting in individual-specific patterns of circadian disruption. It is noteworthy that Wang et al. [5] described abnormalities in the circadian rhythms of stroke patients which were quantitatively dependent on the distance of the infarct from the hypothalamus. Finally, novel genetic polymorphisms in outbred Wistar rats may impact the responses of SCN neurons to cortical and basal ganglial injury.

In addition to the SCN, PVN and IML are important brain structures controlling melatonin production. Only one rat (ID2658) showed lesion near the SCN, whereas none of the MCAo treated rats showed detectable injury in the PVN region. MCAo surgery is known to spare the spinal cord where IML is found. Thus, direct neuronal defects in melatonin rhythm-generating machinery in the brain can be ruled out as the cause of the melatonin rhythm disruptions seen in our studies. Since brain structures such as IGL [20] and Raphe nuclei send projections to the SCN, injury in these regions could potentially affect melatonin output. Upon careful examination, however, none of the rats had lesions in the Raphe nuclei and only three rats (ID2658, ID2639, and ID2896) showed lesion near the IGL. There were no common features of melatonin shifts in rats with IGL lesions compared to those with intact IGL. Previous study also demonstrated involvement of CPu and parietal cortex in locomotor rhythm regulation that can be dissociated from the SCN [21]. In the same study, melatonin rhythms were shown to be phase aligned with the SCN clock gene expression and were not affected by the free-running locomotor rhythms induced by drug treatment [21]. MCAo treated rats in our cohort showed lesions in both CPu (11 of 11 rats) and parietal cortex (8 of 11), two structures that are most consistently affected in this model of stroke. These results suggest that stroke may unmask interactions between the parietal/CPu locomotor rhythm generating system and other SCN-driven rhythms by yet unidentified mechanisms.

Our studies confirm the influence of the cortex and basal ganglia on circadian rhythms. In fact, extrahypothalamic mechanisms of circadian disruption have been described in preclinical models of Huntington's disease [22] and Alzheimer's disease [23]. Thus, uncovering the mechanisms of how brain dysfunction outside the retinohypothalamic pathway impacts circadian timekeeping may ultimately lead to strategies to reduce the morbidity of many neurological disorders. Our model of measuring pineal melatonin after stroke may be useful in deciphering these mechanisms, since it is quantitative and capable of detecting longitudinal changes in the same animals.

## Competing interests

The authors declare that they have no competing interests.

## Authors' contributions

HM designed and performed stroke surgery and performed analysis of circadian and stroke data. TL performed microdialysis surgery and melatonin measurements and analyzed melatonin data. JB directed the melatonin measurement studies, analyzed microdialysis data, edited the manuscript, and designed the figures. MMW directed the stroke experiments, analyzed data, and wrote the manuscript. All authors read and approved the final version of this article.
